# Role of FGF23 in Pediatric Hypercalciuria

**DOI:** 10.1155/2017/3781525

**Published:** 2017-12-31

**Authors:** Maria Goretti Moreira Guimarães Penido, Marcelo de Sousa Tavares, Uri Saggie Alon

**Affiliations:** ^1^Pediatric Nephrology Unit, Clinics Hospital, School of Medicine, Federal University of Minas Gerais, Belo Horizonte, MG, Brazil; ^2^Pediatric Nephrology Unit, The Nephrology Center, Santa Casa de Belo Horizonte Hospital, Belo Horizonte, MG, Brazil; ^3^Bone and Mineral Disorders Clinic, Section of Pediatric Nephrology, Children's Mercy Hospital and Clinics, University of Missouri at Kansas City, Kansas City, MO, USA

## Abstract

**Background:**

This study explored the possible role of FGF23 in pediatric hypercalciuria.

**Methods:**

Plasma FGF23 was measured in 29 controls and 58 children and adolescents with hypercalciuria: 24 before treatment (Pre-Treated) and 34 after 6 months of treatment (Treated). Hypercalciuric patients also measured serum PTH hormone, 25(OH)vitD, phosphate, calcium, creatinine, and 24 h urine calcium, phosphate, and creatinine.

**Results:**

There were no differences in age, gender, ethnicity, or body mass index either between controls and patients, or between Pre-Treated and Treated patients. Median plasma FGF23 in controls was 72 compared with all patients, 58 RU/mL (*p* = 0.0019). However, whereas FGF23 in Pre-Treated patients, 73 RU/mL, was not different from controls, in Treated patients it was 50 RU/mL, significantly lower than in both controls (*p* < 0.0001) and Pre-Treated patients (*p* = 0.02). In all patients, there was a correlation between FGF23 and urinary calcium (*r* = 0.325; *p* = 0.0014). Treated patients had significantly lower urinary calcium (*p* < 0.0001), higher TP/GFR (*p* < 0.001), and higher serum phosphate (*p* = 0.007) versus Pre-Treated patients.

**Conclusions:**

Pharmacological treatment of hypercalciuric patients resulted in significantly lower urinary calcium excretion, lower serum FGF23, and elevated TP/GFR and serum phosphate concentration, without significant changes in PTH. Further studies are indicated. This trial is registered with Clinical Registration Number RBR 8W27X5.

## 1. Introduction

Hypercalciuria is the most common metabolic condition associated with pediatric urolithiasis and some patients demonstrate reduction in bone mass [1–4]. Various mechanisms have been proposed to explain the generalized dysregulation of calcium homeostasis in hypercalciuria, including decreased renal calcium reabsorption, increased gut calcium absorption, and increased bone resorption, or any combination of the above, due to either genetic or environmental effects [5–10].

Studies of adult hypercalciuric stone formers have provided evidence that some of them have significantly lower threshold of tubular reabsorption of phosphate (TP/GFR) and serum phosphorus levels than normal individuals, in the face of normal parathyroid hormone levels [5, 11, 12]. The hypophosphatemia stimulates 1,25(OH)_2_ vitamin D production and consequently hypercalciuria. However, the cause for the altered tubular phosphate reabsorption is not known. It was suggested that variations in the klotho-FGF23 axis could mediate alterations in phosphate handling by the kidney and thus play a role in hypercalciuria, and indeed a few studies in adult calcium stone formers reported higher fibroblast growth factor 23 (FGF23) levels [5, 13, 14]. On the other hand, a recent study could not implicate FGF23 in the bone alterations seen in these patients [15].

Based on these observations, the aim of this study was to explore the possible role of FGF23 in children with hypercalciuria.

## 2. Materials and Methods

We performed a cross-sectional study on 58 children, adolescents, and young adults (0 to 24 years) with hypercalciuria and 29 healthy controls (0 to 24 years). Inclusion criteria for hypercalciuric patients were urinary calcium excretion ≥ 4 mg/kg/24 h, normocalcemia, and diurnal and nocturnal continence. The control group consisted of sex- and age-matched healthy subjects from our Pediatric Primary Care Center. Health status was determined through the subjects' medical history and either a parental report or self-report and clinical examination. In all participants, exclusion criteria included nephrocalcinosis, chronic immobilization, hypercalcemia, history of malignancy, excessive oral ingestion of calcium or vitamin D, hyperuricemia, long-term steroid treatment, history of other drug use with possible actions on calcium and phosphorus metabolism during the preceding 2 years, and concurrent conditions associated with metabolic bone disease or renal tubular defects. Of the hypercalciuric patients, 24 were evaluated while receiving no treatment (Pre-Treated group) and 34 patients were evaluated after 6 months of treatment (Treated group). The latter were treated with hydrochlorothiazide 0.5–1.0 mg/Kg/day. Thiazide treatment was initiated when there were low bone mineral density and/or fractures, and no improvement of calciuria levels and symptoms was obtained with the initial approach (dietary modification with high fluid intake, low sodium, and the RDA of protein and calcium) [16].

All hypercalciuric patients were asked to continue their daily routine in terms of diet, physical activities, and sun exposure during the blood and urine sample collections. A 24-hour urine sample collected at home and an overnight fasting venous blood sample were obtained at the hospital laboratory. Collection accuracy was verified by urine creatinine excretion rate [17]. The urine sample was analyzed for volume, creatinine, calcium, and phosphate. The blood sample was analyzed for creatinine, calcium, phosphorus, parathyroid hormone (PTH), 25(OH) vitamin D [25(OH)D], and FGF23. Clinical data included age at diagnosis, gender, ethnicity, body mass index (BMI), and history of urolithiasis. In the healthy controls, a blood sample for FGF23 was obtained in all 29. In 16 of them, 24-hour urine calcium, phosphate, and creatinine were analyzed as part of their evaluation for voiding dysfunction.

Analysis of blood and urine phosphate, calcium, creatinine, 25(OH)D, and intact PTH was conducted using standard laboratory methods. Plasma FGF23 concentrations were assessed using a C-terminal assay-ELISA (Immutopics International, San Clemente, CA, USA).

Statistical analysis was performed with SPSS 20 version 18.0 (SPSS, Chicago, IL) software for Windows. The normality of data distribution was analyzed using Shapiro-Wilk's test. Accordingly, descriptive data are expressed as mean ± SD or median (25th and 75th percentiles). Differences between groups were analyzed using two-tailed *t*-test for unpaired samples when normality conditions were fulfilled and the Wilcoxon rank sum test (two-sided) or Kruskal-Wallis test when they were not. ANOVA was applied for comparisons between three or more groups. Correlations between blood and urinary biochemical parameters were assessed by Spearman correlation test. The level of significance was set up as *p* < 0.05.

The study was approved by the institutional review board of the Clinics Hospital of the Federal University of Minas Gerais, Brazil (ETIC0479.0.203.000-10, December 01, 2010), and was performed in accordance with the ethical standards laid down in the 1964 Declaration of Helsinki. The participants and/or their guardians signed informed consent forms.

## 3. Results

Demographic and clinical data of the controls, patients, and their subgroups are shown in Table 1. There were no differences in age, gender, BMI* Z*-score, or ethnicity between controls and all hypercalciuric patients, or between the Pre-Treated and Treated patients. There was no difference in the incidence of urolithiasis between the Pre-Treated and Treated patients.

As shown in Table 2, the median value of FGF23 in all hypercalciuric patients, 58 RU/mL (7–368), was statistically lower than 72 RU/mL (56–210) in controls (*p* = 0.0019). Further analysis showed that whereas the median value of FGF23 in Pre-Treated patients, 73 RU/mL (32–190), was not different than in controls, that of Treated patients, 50 RU/mL (7.00–368), was significantly lower than in both, controls (*p* = 0.0001) and Pre-Treated patients (*p* < 0.02).

Blood and urine data from Pre-Treated and Treated hypercalciuric patients are shown in Table 3. There were no differences between the Pre-Treated and Treated hypercalciuric patients regarding serum creatinine, calcium, 25(OH)D, PTH, urine creatinine, and urine phosphate. Treated patients had significantly lower urinary calcium (*p* < 0.0001), higher TP/GFR (*p* = 0.0018), and higher serum phosphate (*p* = 0.007) when compared to Pre-Treated patients.

The 24-hour urine calcium excretion in the 16 controls of 2.50 ± 1.02 mg/kg was not different from the Treated group 2.51 ± 0.71 (*p* = 0.739), but significantly lower than in the Pre-Treated group, 5.63 ± 1.21 (*p* < 0.001).

The 24-hour urine phosphate excretion (mg/kg) was not different amongst the three groups, controls (15.76 ± 9.31), Pre-Treated group (16.41 ± 5.61), and Treated group (13.84 ± 4.55).

**Figure 1:**
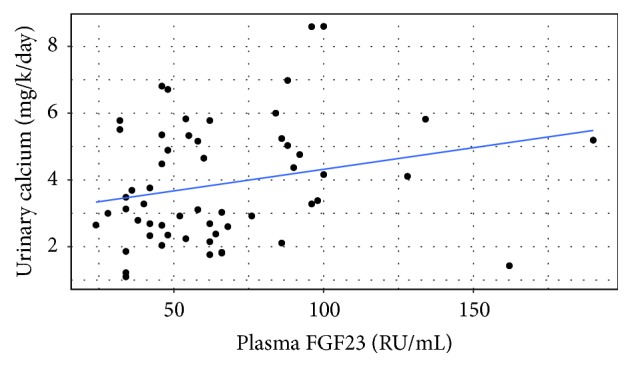
Correlation between 24-hour urinary calcium (mg/Kg) and serum FGF23 in hypercalciuric patients.

As depicted in Figure 1, in the hypercalciuric children, a positive correlation was found between 24-hour urine calcium and serum FGF23 (*r* = 0.325; *p* = 0.0014).

## 4. Discussion

FGF23, calcitriol, and PTH are all involved in mineral metabolism in the gut, bone, and kidney with the goal to maintain optimal calcium and phosphate homeostasis [5, 18, 19]. Thus, these regulatory hormones may play a role in urine calcium excretion and consequently pathogenesis of hypercalciuria [13]. In the present study, we explored the possible role of FGF23 in pediatric hypercalciuria.

We found that the median value of FGF23 in Pre-Treated patients was not different than in controls (Table 2). Higher levels of FGF23 were previously reported in some adult patients with calcium stones [13, 14]. Rendina et al. found that, out of 110 calcium stone formers, 22 demonstrated renal phosphate leak and significantly higher FGF23 levels compared with controls and with the other 88 stone formers without renal phosphate leak [13]. Taylor et al. in a prospective study found a trend for higher plasma FGF23 concentration in symptomatic men with kidney stones, which however did not reach statistical significance [14]. It thus seems that the normal mean serum FGF23 value observed in our Pre-Treated group might be due to the fact that none were hypophosphatemic (Table 2).

Our hypercalciuric patients demonstrated a positive correlation between 24-hour urinary calcium and serum FGF23 (Figure 1). However the current study cannot indicate direct cause and effect relationship between the two. Repeat observations in adult hypercalciuric stone formers showed elevated serum 1,25(OH)_2_D_3_ concentration [14, 20–22]. Furthermore it has been demonstrated that the administration of calcitriol to normal men replicates characteristic features of hypercalciuria [23, 24]. It is thus possible that 1,25(OH)_2_D_3_ is the main regulator of the observed concomitant changes in FGF23 and urinary calcium; namely, when 1,25(OH)_2_D_3_ is high both plasma FGF23 and urine calcium increase and vice versa. Indeed, in patients with genetic hypophosphatemic rickets due to high circulating serum FGF23 levels, neither 1,25(OH)_2_D_3_ nor urine calcium is elevated; namely, high levels of FGF23 per se do not cause hypercalciuria [19].

Previous studies, including ours, demonstrated decreased bone mineral density in adult and pediatric hypercalciuric patients [2–4]. However, the underlying pathophysiologic mechanism for the altered bone remodeling process in hypercalciuric patients remains unknown. Considering that FGF23 is an osteocyte-derived hormone, it is possible that higher levels in stone formers could be a marker of abnormal bone physiology. However, Barcellos Menon et al. [15] recently showed that FGF23 expression in osteocytes from hypercalciuric patients was not different from controls and did not correlate with histomorphometric parameters. On the other hand, these researchers found that bone resorption in these patients could be implicated on 1,25(OH)_2_D with no correlation with serum PTH [15]. Similarly, the TP/GFR and serum phosphate decreased in the face of equal mean values of PTH, thus, more likely implicating FGF23 (Table 3).

We therefore propose that different or additional mechanisms are involved in hypercalciuria including environmental ones and in particular the effect of nutrition [25]. It has recently been shown that the use of the DASH diet in hypertensive adults results in decreased incidence in urolithiasis [26]. Similar changes were observed in children; namely, decreased dietary intake of sodium on one hand and increase in potassium intake on the other led to a decrease in calciuria and stone formation [7–9].

Following anticalciuric treatment, the urine calcium excretion in our patients normalized to the level of healthy controls (Table 3). With that, serum FGF23 decreased and, as expected, both TP/GFR and serum phosphate increased. It thus seems that, with improved calcium retention, a series of unknown events took place resulting in lower plasma FGF23 concentration. As discussed earlier, the change could have been mediated by decrease in serum 1,25(OH)_2_D_3_ concentration or activity on the bone [15, 27].

Our study has some limitations. Unfortunately due to budgetary constraints, serum 1,25(OH)_2_D_3_ concentration was not measured. Changes in its level before and following anticalciuric treatment may have served as the missing link in explaining the phenomena observed. Availability of blood and urine data in the control group may have disclosed discrepancies compared with the findings observed in the hypercalciuric patients. The lack of any data on 1,25(OH)_2_D_3_ levels is a weakness in our study as well as the lack of serum PTH, creatinine, calcium, and phosphate values in controls. Nevertheless, we believe that the aforementioned limitations do not interfere with the observations made in this study of the likelihood of lack of direct role of FGF23 in pediatric hypercalciuria on one hand and the changes observed in its metabolism once pharmacologic anticalciuric intervention has been introduced on the other hand.

In conclusion, our study found no difference in plasma FGF23 levels between hypercalciuric and control children. Pharmacologically treated patients had significantly lower urine calcium excretion rate, lower plasma FGF23 levels, and elevated TP/GFR and serum phosphate without significant changes in serum PTH values. It thus seems that the reversal of hypercalciuria may directly or indirectly affect phosphate metabolism. We recommend that future studies will differentiate between Treated and Pre-Treated patients and will include 1,25(OH)_2_D_3_ amongst all other measured variables.

## Figures and Tables

**Table 1:** Demographic data of hypercalciuric (H) patients, controls, and Pre-Treated and Treated patients with hypercalciuria.

Parameters	H patients *N* = 58	Controls *N* = 29	*p*	Pre-Treated *N* = 24	Treated *N* = 34	*p*
Age (yr)	16.3 (6.3–24.8)	16.7 (2.0–24.7)	0.655	16.9 (7.6–24.8)	15.7 (6.3–23.7)	0.33
Male (*n*/%)	35/60.5	20/66.5	0.561	14/58.5	21/62	0.79
BMI *Z*-score	−0.19 ± 1.26	−0.35 ± 1.48	0.688	−0.02 ± 1.24	−0.29 ± 1.29	0.49
White skin color (*n*/%)	38/65.5	17/57		13/54	23/67.5	
Urolithiasis (Y/%)	44/76	0/0	-	18/75	26/76.5	0.89

Data are median (25th and 75th percentiles), mean ± SD, or percentage (number); Y: yes; tests: two-tailed *t*-test, Wilcoxon rank sum test (two-sided), or Kruskal-Wallis test, ANOVA.

**Table 2:** FGF23 (RU/mL) of controls, hypercalciuric (H) patients, and Pre-Treated and Treated patients with hypercalciuria.

	Median (min–max)
Controls (*n* = 29)	72.0(56.0–210.0)^*∗*●#^
H patients (*n* = 58)	58.0(7.0–368.0)^*∗*^
Pre-Treated (*n* = 24)	73.0(32.0–190.0)^*◆*#^
Treated (*n* = 34)	50.0(7.0–368.0)^*◆*●^

^*∗*^ *p* = 0.009; ^*◆*^*p* = 0.02; ^●^*p* < 0.0001; ^#^*p* = 0.26; test: Wilcoxon rank sum test (two-sided).

**Table 3:** Blood and urine biochemical parameters in Pre-Treated and Treated patients with hypercalciuria (mean ± SD).

	Pre-Treated *n* = 24	Treated *n* = 34	*p*
Blood			
Creatinine (mg/dl)	0.74 ± 0.04	0.67 ± 0.03	0.22
Calcium (mg/dl)	9.59 ± 0.50	9.70 ± 0.46	0.35
Phosphate (mg/dl)	4.01 ± 0.60	4.44 ± 0.49	*0.007*
PTH (pg/ml)	29.94 ± 12.25	25.90 ± 11.21	0.28
25(OH)D (ng/ml)	28.71 ± 6.25	32.32 ± 7.86	0.11
24-hour urine			
Creatinine (mg/kg)	21.9 ± 5.1	20.1 ± 3.3	0.24
Calcium (mg/kg)	5.63 ± 1.21	2.51 ± 0.71	*<0.001*
TP/GFR	3.42 ± 0.15	3.97 ± 0.10	*0.001*

Test: Wilcoxon scores and Student's *t*-test; TP/GFR: phosphate tubular reabsorption by glomerular filtration rate; 25(OH)D: 25OH vitamin D; PTH: parathyroid hormone.
